# Ecological and Human Health Risks from Potentially Toxic Elements in Environmental Matrices of Kiteezi Landfill, Uganda

**DOI:** 10.3390/jox15060185

**Published:** 2025-11-04

**Authors:** Emmanuel Ebbu, Irene Nalumansi, Ivan Kiganda, Caroline Kiwanuka Nakiguli, Patrick Onen, Simon Ocakacon, Christopher Adaku, Timothy Omara, Emmanuel Ntambi

**Affiliations:** 1 Department of Chemistry, Faculty of Science, Mbarara University of Science and Technology, Mbarara P.O. Box 1410, Uganda; ebbuemma@yahoo.com (E.E.); inalumansi@must.ac.ug (I.N.); cnakiguli@must.ac.ug (C.K.N.); cadaku@must.ac.ug (C.A.); 2 Department of Chemistry, College of Natural Sciences, Makerere University, Kampala P.O. Box 7062, Uganda; ivan.kiganda@gmail.com; 3 Department of Chemistry, Faculty of Science, Kyambogo University, Kampala P.O. Box 1, Uganda; patrickonen1995@gmail.com; 4 Department of Civil and Environmental Engineering, College of Engineering, Design, Art and Technology, Makerere University, Kampala P.O. Box 7062, Uganda; ocakaconsimon@gmail.com

**Keywords:** bioaccumulation, Kitetikka stream, municipal solid waste, toxic element

## Abstract

By the time of this study, Kiteezi landfill was Uganda’s largest waste disposal site and received substantial volumes of municipal solid waste. In the present study, water (*n* = 36), leachates (*n* = 36), superficial sediments (*n* = 30), and *Colocasia esculenta* corms (*n* = 6) were sampled from Kiteezi landfill in the dry and wet seasons of 2022 before its tragic collapse in 2024. The physicochemical parameters (pH, electrical conductivity, temperature, and oxidation–reduction potential) and concentration of potentially toxic elements (As, Cu, Cr, Pb, and Zn) were analyzed using standard methods and inductively coupled plasma-optical emission spectrometry, respectively. Significant seasonal variations (*p* < 0.05) were observed for all the physicochemical parameters of water and leachates except temperature. Further, significantly higher concentrations (*p* < 0.05) of potentially toxic elements (PTXEs) were quantified in environmental matrices sampled during the dry season than the wet season. Arsenic and Pb concentrations in water surpassed their WHO permissible limit of 0.01 mg/L. The concentrations of PTXEs were higher in downstream samples (*p* < 0.05), indicating that landfill activities led to their enrichment in matrices near the facility. Ecological and pollution risk indices indicated that there is severe enrichment of Cu and Zn in the sediments, with dry season downstream samples having contamination factors and geoaccumulation indices of 539.3 and 74.7 and 8.5 and 5.6, respectively. Although ingestion of water may not cause probable health risks, consumption of *Colocasia esculenta* corms could lead to non-carcinogenic and cancer health risks in both children and adults (hazard indices = 0.085–189.0 and total cancer risk values of 7.33 × 10^−6^–4.87 × 10^−3^). These results emphasize the need that any new replacement for Kiteezi landfill should be properly planned and managed to mitigate potential environmental pollution with xenobiotics.

## 1. Introduction

Environmental pollution is one of the major challenges impeding the realization of the Sustainable Development Goals (SDGs) [1]. This is especially true because the SDGs take a nexus approach, and those goals related to poverty (SDG 1), clean water and sanitation (SDG 6), good health and wellbeing (SDG 3), sustainable cities and communities (SDG 11), life below water (SDG 14), and life on land (SDG 15) are directly affected by pollution [2]. For example, air pollution, which is responsible for close to 7 million mortalities annually, is now listed as the biggest environmental health risk of our time [3]. Post COVID-19 pandemic, global discourse on waste management has marked it as central to addressing the interconnected challenges of climate change, pollution, and biodiversity loss [4].

Effective waste management strategies are essential for mitigating greenhouse gas emissions, reducing environmental contamination, and protecting ecosystems. Landfilling remains one of the most widely used waste management methods, yet it is linked to several environmental pollution issues such as contamination of groundwater due to the leaching of complex mixtures of contaminants in waste, air pollution from the suspension of particulate matter, and odor from putrefying wastes [5]. Together, these can directly or indirectly impact public health, environmental quality, and livelihoods [6,7]. In this context, pollution from landfills by potentially toxic elements (PTXEs) is a growing global concern due to the persistence, bioaccumulation, and potential toxic effects of these contaminants to ecosystems and humans [8]. The situation has worsened in developing countries because rapid urbanization and industrialization have considerably increased waste generation, yet the corresponding waste management infrastructure cannot sufficiently cope with the increasing waste volumes [9,10].

The present study focused on Kiteezi landfill (KTLF) of Uganda, which was by the time of this study the country’s premier and largest waste disposal site [6,11]. The landfill had malfunctioning leachate treatment facilities, which allowed contaminants to infiltrate soil, ground, and surface water [12]. Given the indiscriminate disposal of industrial, electronic, and domestic waste, the site served as a potential hotspot for the accumulation of PTXEs. Earlier research reported a high concentration of PTXEs such as copper (Cu), cadmium (Cd), chromium (Cr), lead (Pb), and zinc (Zn) in leachates, water, and grasses around KTLF [13,14,15,16]. Leachates from KTLF may also percolate into nearby water sources, leading to pollution that poses risks to local communities relying on these water resources for domestic use, irrigation, and aquaculture [12,13,14,16,17,18]. The PTXEs in water can become adsorbed onto sediments, where they bind to organic and inorganic matter, creating long-term contamination reservoirs. Over time, such PTXEs can be released back into the water column due to changes in environmental conditions, leading to secondary contamination and increased bioavailability [19]. Another concern could be the bioaccumulation of PTXEs in aquatic organisms in nearby streams and edible plants such as taro (*Colocasia esculenta* (L.) Schott) which are cultivated in the vicinity of KTLF.

Despite previous studies on landfill leachate and environmental contamination in KTLF [12,13,14,16,17,18,20], limited information exists on the temporal variations in the physicochemical properties of water and leachates as well as cross-matrix (leachate–water–sediment–vegetable) distribution of PTXEs in and around KTLF. The present study provides the first ever integrated assessment of PTXEs in leachates, surface water, superficial sediments, and *Colocasia esculenta* (*C. esculenta* henceforth) from KTLF, the largest and historically most important waste disposal site in Uganda. Unlike earlier studies which investigated individual matrices or general pollution levels, the present study combined elemental analysis with ecological and human health risk assessment models to identify exposure pathways and risk magnitudes across the different environmental compartments.

## 2. Materials and Methods

### 2.1. Description of the Study Area and Reconnaissance Survey

**Figure 1:**
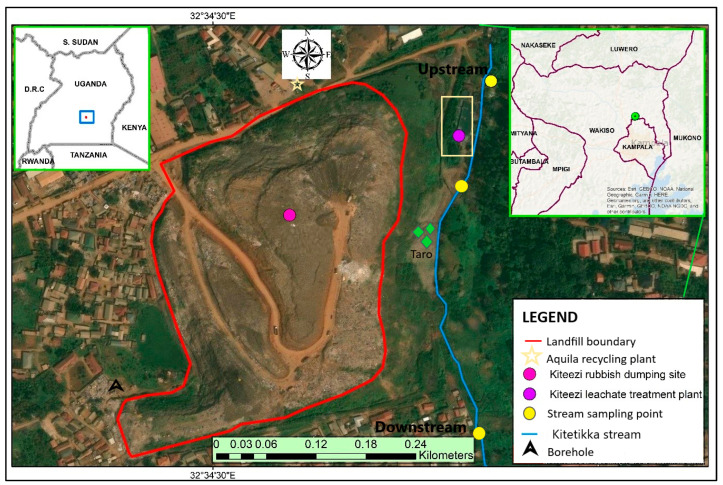
Map showing the location of the Kiteezi landfill and the sampled compartments. Inset is the location of the landfill in Uganda (upper left) and Wakiso district (upper right). The second borehole considered in the present study is located about 1 km east of the Leachate Treatment Plant, previously sampled by Aryampa et al. [12]. Parallel clarifiers 1 and 2 connect to the Kitetikka stream about 5 m before where downstream samples were taken. Map was adapted from Google Maps [22] and prepared in ArcGIS (version 11.5).

**Figure 2:**
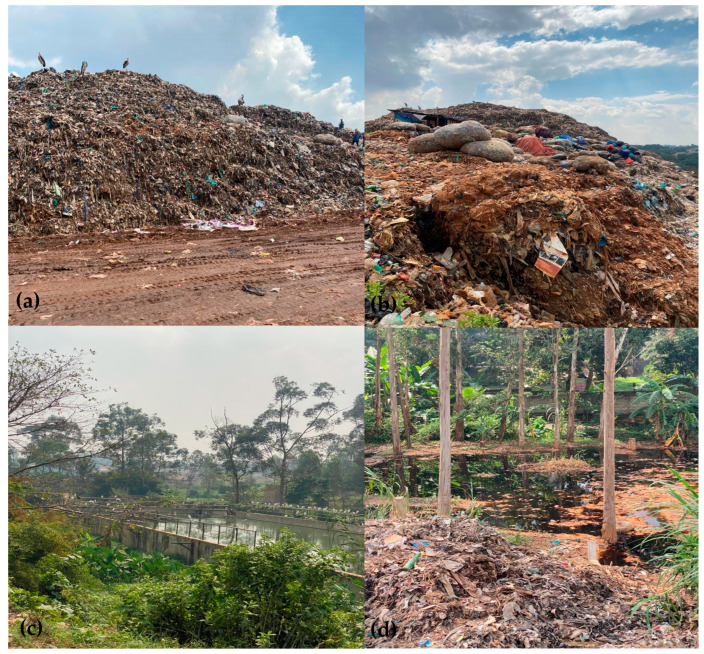
An overview of Kiteezi landfill and its sampled compartments: (**a**,**b**) depict piles of waste with marabou storks (*Leptoptilos crumenifer*) and waste scavengers on the piles; (**c**) leachate management pond with taro plants (*Colocasia esculenta*) in the left middle ground and white little egrets (*Egretta garzetta* L.) standing on rails in the far background; and (**d**) garbage and leachate leakage onto nearby farms with cultivated food crops.

Located in Mpererwe, KTLF (0°24′39.0″ N, 32°34′33.0″ E; Figure 1) was the only recognized landfill receiving waste from Kampala City at the time of this study. The facility is situated in Kiteezi village, about 15 km from Kampala City, and it was owned and managed by Kampala Capital City Authority (KCCA). The landfill was opened in 1996, with a total land coverage of 35–36 acres [15]. It received approximately 1300 tons of waste/day, a volume that grew beyond its earlier daily capacity of 946 tons/day [21]. The waste composition of KTLF was dominated by organic materials (such as food waste, market residuals, and vegetation), which constitute up to 90% of the total wastes. Plastics constituted at least 3.7%, whereas paper and packaging items, textiles, and broken glass accounted for 1.62%, 0.50%, and 0.68%, respectively [16,21]. Thus, KTLF represented an interesting study area due to the diversity and composition of the waste it received from Kampala City (Figure 2).

Further, KTLF was established on a reclaimed wetland (that is, an open space with partial fencing) [21]. Thus, the landfill ends in a wetland through which the Kitetikka stream runs, and this stream feeds some of the community water supply [16,18]. The landfill has habitation and animal farms within a less than 500 m radius [11,16]. Such a setting, from a toxicological standpoint, should expose the residents to fetid odor and other toxicants arising from the burning of plastics. To the eastern boundary of KTLF is a seasonal wetland (Walufumbe swamp positioned along River Walufumbe [23]), and it empties its water into the Victoria Nile catchment.

The KTLF area experiences a tropical equatorial climate characterized by bimodal rainfall patterns. The long rainy season occurs from March to June, followed by a shorter one between November and December, although interannual variability is common. The region receives an average annual rainfall of 1300 mm, with mean daily maximum and minimum temperatures of 28 °C and 16.5 °C, respectively [12]. The KTLF reached its maximum capacity in 2012, and instead of it being decommissioned or expanded, the sanitary engineered landfill continued to operate, creating a pile of waste (Figure 2). On Friday, 9 August 2024, a devastating collapse of the pile occurred in which at least 35 people died and 1000 others were displaced [24]. This tragic avalanche followed torrential rains which have been anecdotally attributed to the direct impact of climate change, although methane explosion could be another possible trigger.

Prior to this study, a reconnaissance survey was conducted. It was observed that waste was covered regularly with excavated soils (murram), and this had made the site a “hill-shaped” structure. At the base/bottom of the structure are two parallel drainage channels, which discharge leachate into the stream, and another channel that discharges leachate into a clarifier in the Leachate Treatment Plant (LTP) [12]. Leachate from KTLF flows through open channels (trenches) and is directed by 160 mm high-density polyethylene pipes to the temporary leachate collection pit, which is transported to the LTP on the northeastern part of the landfill. The quantity of raw leachate directed to the LTP is about 166 m^3^/day [25], but the plant was malfunctional at the time, and leachates were being directly discharged into Kitetikka stream.

Different land-use practices were noted around the KTLF area, which contains alluvial soil [26]. Wetland, bushes, wild tomatoes, *Amaranthus* species, trees (*Cyperus papyrus*, *C. rotundus*, *Eleis guinensis*, *Phoenix reclinata*, *Phragmits australis*, *Leersia hexandra*, *Ludwigia abyssinica*, *Imperata cylindrica*, and *Rhynchelytrum repens*), and *C. esculenta* on the eastern boundary of the site were the key sensitive receptors [16,21]. Birds such as marabou storks (*Leptoptilos crumenifer*), crested cranes (*Balearica regulorum*), little egrets (*Egretta garzetta* L.), hadada ibis (*Bostrychia hagedash*), and pied crows (*Corvus albus*) previously sited at KTLF [23] were also spotted (see Figure 2).

### 2.2. Sampling of Environmental Matrices

Some environmental factors such as lithology, hydrology, climate, seasons, and land use were considered because they contribute to the transit of PTXEs. Therefore, sampling was conducted in three different receiving environments namely: stream water, sediments, and the LTP. This was performed in triplicate during the dry and wet seasons (June 2022 and August 2022).

Surface water samples from the nearby stream were collected using dip samplers and transferred into 100 mL polystyrene bottles. From the point of leachate discharge, grab samples were taken from approximately 500 m upstream (*n* = 12) and then downstream (*n* = 12; see Figure 1). Groundwater samples were taken from a borehole located adjacent to the landfill (western arm; *n* = 6) and another borehole located northeast of the LTP (previously sampled by Aryampa et al. [12]; *n* = 6). Then, a prepared 1% nitric acid solution was added to each sample collected to ensure that the pH drops below 2.

Superficial sediments were sampled from the two leachate channels (*n* = 12), the leachate clarifier (*n* = 6), upstream (*n* = 6) and downstream (*n* = 6), using a grab sampler at a depth of 0–5 cm [27]. Leachates were sampled by scooping raw leachates along the two main channels (*n* = 24) and the final effluent (clarifier) at the LTP (*n* = 12). Sampling of *C. esculenta*, which is an edible root vegetable grown along the stream, was performed by harvesting the plants and cutting out the corms (*n* = 6).

### 2.3. Determination of Non-Conservable Parameters of Leachate and Water Samples

We determined the pH, electrical conductivity, oxidation–reduction potential, and temperature of the leachate and water samples in situ following standard methods [28,29].

### 2.4. Sample Preparation Procedures

Sediments and *C. esculenta* corms were prepared following the microwave-assisted acid digestion method used previously [30,31]. Briefly, 1 mg of finely crushed solid sample was digested with 1 mL of concentrated HNO_3_ and 3 mL of concentrated HCl in an ETHOS Milestone microwave digester (Milestone™ Srl, Sorisole, Italy). The vessels/cells were subjected to 200 °C and 110 psi for 15 min. A measured 1 mL of the digested sample was diluted with 100 mL of double distilled water. For water and leachates, the acidified samples were first filtered and then analyzed for their dissolved PTXE contents, in the same way as for the sediments and *C. esculenta*.

### 2.5. Instrumental Analysis, Quality Assurance, and Quality Control

The PTXEs (As, Cu, Cr, Pb, and Zn) in the sample solutions were quantified spectrometrically using an inductively coupled plasma-optical emission spectrometer (ICP-OES; Optima 7000 DV, Perkin Elmer Inc., Waltham, MA, USA) as described previously [32,33]. For this purpose, the specific wavelengths used for the PTXEs were 193.696, 214.440, 224.700, 267.716, 220.353, and 206.200 nm, respectively. Quantitative analysis was based on five-point external calibration curves constructed from working standard solutions diluted from 1000 mg/L stock solutions of the respective metal nitrate or chloride [32].

As a quality assurance measure, only analytical-grade reagents and chemicals were used for the digestion and analysis of samples. The analytical quality control measure involved the analysis of procedural blanks and spiked samples for every 10 samples. The recoveries for spiked samples ranged from 99.7 to 100.5%. Throughout the analyses, batch-specific errors were prevented through the analysis of all samples in triplicate under the same standard conditions. We also computed method precision as relative standard deviations, which fell below 5% (3.8–4.6%).

### 2.6. Dietary Exposure to PTXEs and the Associated Human Health Risks

Health risks associated with ingestion of water and consumption of *C. esculenta* corms were determined as cancer and non-cancer risks for both children and adults. Assessing these risks requires determining the estimated daily intake (EDI) of the PTXEs (Equation (1)) [27]:

(1) $$\mathrm{EDI}=\frac{C_{hm}\;\times \;F_{i}\;\times \;ExFr\times \;ExDr}{W_{b}\;\times \;T_{a}}$$

where C*_hm_* = measured PTXE concentration, F*i* = ingestion rate for water or *C. esculenta* corms, E*_X_*F*r* = exposure frequency, E*_X_*D*r* = exposure duration, $W_{ab}\;$= body weight, and T*a* = average time (Table S1).

On the one hand, non-carcinogenic health risk was estimated following the hazard quotient (HQ) method (Equation (2)). Since PTXEs exhibit additive toxicity, the hazard index (HI) was calculated by summing up the HQ of the individual PTXEs (Equation (3)) [34]:

(2) $$\mathrm{HQ}\;=\;\frac{EDI}{RF}$$

(3) $$\mathrm{HI}=\sum_{i}^{n}HQ$$

Here, RF is the oral reference dose of the heavy metal, with values of 0.0003, 0.04, 0.001, 0.0014, and 0.006 mg/kg for As, Cu, Cr, Pb, and Zn, respectively [34,35].

On the other hand, carcinogenic risk (CR) was calculated using the incremental lifetime cancer risk for ingestion of carcinogenic PTXEs (As, Cr, and Pb) in water and *C. esculenta* corms (Equation (4)) [36]. The total cancer risk (TCR) through an exposure pathway in each case was taken as the sum of the CR posed by each carcinogenic element (Equation (5)):

CR = EDI × IGCSF(4)

(5) $$\mathrm{TCR}=\sum_{1}^{4}CR$$

where the ingestion cancer slope factor (IGCSF) = 1.5 × 10^0^, 5.0 × 10^−4^ and 8.5 × 10^−3^ mg/kg/day for As, Cr, and Pb [36].

### 2.7. Evaluation of Sediment Pollution Levels

Sediment quality was assessed by calculating the contamination factor (CF) and geoaccumulation index (I_geo_) using Equations (6) and (7) [37,38]:

(6) $$\mathrm{CF}\;=\;\frac{C_{hm}}{C_{P}}$$

(7) $$I_{\mathrm{geo}}={\mathrm{Log}}_{2}\;\frac{C_{hm}}{{1.5\;C}_{P}}$$

Here, C*_hm_* is the measured PTXEs’ concentration, and C*_P_* refers to the pre-industrial concentration of the PTXEs (1.8, 12.5, 60, 55, and 70 mg/kg for As, Cu, Cr, Pb, and Zn, respectively, in crustal shale [39]). The constant (1.5) is a pure background matrix correction factor, introduced to account for expected lithological variations [32,38].

To estimate the overall pollution burden in superficial sediments, the pollution load index was calculated as follows (Equation (8)) [34]:

Pollution Load Index (PLI) = (CF*_As_* × CF*_Cu_* × CF*_Cr_* × CF*_Pb_* × CF*_Zn_*)^1/5^ (8)

The indices CF*_As_* to CF*_Zn_* are the contamination factors for the PTXEs, which follows from Equation (6). To evaluate the potential ecological threat posed by the PTXEs to aquatic organisms, the potential ecological risk index (PERI) was used, employing Equations (9) and (10) in tandem [38]:

(9) $$\mathrm{Ecological}\;\mathrm{risk}\;\mathrm{coefficient}\;(E_{R}^{i})\;=\;T_{R}^{i}\times \mathrm{CF}$$

(10) $$\mathrm{Potential}\;\mathrm{ecological}\;\mathrm{risk}\;\mathrm{index}\;(\mathrm{PERI})=\sum_{i=1}^{i=n}E_{R}^{i}$$

The biological toxic factors ($T_{R}^{i}$) are 10, 5, 2, 5, and 1 for As, Cu, Cr, Pb, and Zn, respectively [38]. The interpretation criteria for all the sediment pollution indices calculated are provided in Table S2.

### 2.8. Statistical Analysis

Quantitative data were assessed for normality using the Shapiro–Wilk test. Subsequently, one-way analysis of variance or the Kruskal–Wallis test, as appropriate, was used to determine statistically significant differences among the means of the physicochemical parameters and the PTXEs’ concentrations in a given environmental matrix sampled from the different sites. Tukey’s post hoc or Dunn’s tests were used for multiple comparisons whenever statistical differences were detected at *p* < 0.05. The analyses as well as data visualizations were conducted in Origin Pro 2025b (OriginLab Corporation, Northampton, MA, USA).

## 3. Results and Discussion

### 3.1. Temporal Variations in Water and Leachate Chemistry

The pH of the leachates was the highest (8.1 ± 0.1–9.5 ± 0.1), followed by those of the water sampled from downstream and then water from the borehole (Table 1). There were significant differences among the pH readings of the sampled matrices and between seasons (*p* < 0.05). High pH levels (7.89–8.44) of leachates were reported by a previous study in KTLF, Jinja landfill [20], and Aler dumpsite [40] of Uganda. Other studies in Iran [41], Vietnam [42], India [43], and Greece [44] found comparable pH values of 4.57–8.95, 6.5–9.8, 7.14–8.39, and 4.9–6.7, respectively, for landfill leachates. Nevertheless, alkaline pH is common for leachates from older landfills (at least greater than 10 years old) [43,45]. This increase is expected because the concentration of partially ionized free volatile fatty acids decreases with time [46]. Water from Kitetikka stream and the boreholes had comparatively lower pH levels (5.3–8.5), similar to those reported earlier by Aryampa et al. [12]. These are also comparable to previous observations for the water sources of Aler dumpsite, Uganda (pH = 5.68–6.87) [40], a landfill in Bloemfontein, South Africa (pH = 7.7) [5], and Gaborone sanitary landfill of Botswana (pH = 7.88–8.75) [47]. The pH of borehole water samples were, however, below the acceptable range of 6.5–8.5 initially indicated for potable water by the WHO [48]. Although no health-based guideline value for pH has since been fixed by the WHO from 2022, it should be emphasized that higher pH values can induce unpleasant odor in water and may result in gastrointestinal tract irritation [49].

For electrical conductivity (EC), the values ranged from 233.8 ± 1.20 μS/cm for borehole samples to 27,133.3 ± 1050.40 μS/cm for the leachate samples from Channel 2. There were significant variations in the EC among the sampled matrices and between seasons (*p* < 0.05). Bakyayita et al. [20] reported an average EC of 5100 μS/cm and 3120 μS/cm, respectively, for water from Kiteezi and Jinja landfills of Uganda. Previous studies [12,16] found that the EC of borehole and well water around KTLF ranged from 6.20 to 8.41. For leachates, the ECs reported are higher than 11,345–22,384, 78.6–394.0, 14–133, 10.96–22,570, and 355 µS/cm reported for KTLF [12,16], Aler dumpsite (Uganda) [40], and other landfills in Iran [41], India [43], and South Africa [5].

The high EC values observed in the KTLF leachates could reflect the accumulation of dissolved inorganic ions and salts derived from waste decomposition and oxidation of metallic components, especially electronic waste. Although the analysis of specific ionic species was beyond the scope of the current study, earlier reports on KTLF raw leachates quantified conductive ions (Al^3+^, Ca^2+^, Fe^2+^, K^+^, Mg^2+^, Na^+^, NH_4_^+^, NO_2_^−^, and NO_3_^−^), with NH_4_^+^ and Na^+^ concentrations reaching up to 1554 mg/L and 5245 mg/L [12,16,20]. These species are known to contribute significantly to the EC of landfill leachates [40]. Such ionic enrichment is unmistakable for mature landfill leachates, where long-term waste degradation and leachate recirculation enhance the dissolution of mineral and metallic constituents. Thus, the exceptionally high EC recorded for the leachates could suggest a gradual process of mineralization [50,51].

Temperature measurements across the various sampling points within and around KTLF showed minimal variation, ranging from 20.6 ± 0.4 to 24.6 ± 2.4 °C. One-way ANOVA revealed no statistically significant differences among the sample groups (*p* > 0.05), indicating that temperature remained relatively uniform across both leachate and water sources during the sampling period. The narrow temperature range suggests that external environmental conditions, rather than localized biological or chemical activity, were the dominant influence on the observed values. Bakyayita et al. [20] recorded average temperatures of 27 °C and 30 °C in water sampled from the Kiteezi and Jinja landfills of Uganda. In Nam Son landfill (Vietnam) and municipal waste landfills of Malaysia, temperatures ranged from 20 °C to 33 °C and 25.7 °C to 32.6 °C [42,52], which are higher than those observed in KTLF. Temperature is one of the primary physical properties of water and leachates because it affects other parameters such as color, odor, chemical reaction rates, and the solubility of the entrained compounds. High temperature enhances the growth of pathogenic microorganisms and may increase challenges related to corrosion, taste, odor, and turbidity or color [48].

The oxidation–reduction potential (ORP) varied significantly from −110.0 ± 4.2 mV to 78.5 ± 4.9 mV in the water and leachate samples (*p* < 0.05). The ORP provides an understanding of the redox status of the landfill environment, which governs the mobility, transformation, and persistence of various contaminants. In this study, the ORP values varied markedly across sampling points, indicating dynamic redox conditions within the landfill system. Compared to previous studies, the ORP of leachates was reported to vary from −116.9 mV to −52.1 mV and −53.0 mV to −11.0 mV for landfills in Tripoli, Lebanon [53], and Thessaloniki, Greece [44]. Negative and fluctuating ORP values observed for the leachates during the present study could be linked to the stability and the reduced state of the leachates [44].

The significant temporal variations observed for all the physicochemical parameters of the water and leachate samples except temperature could be attributed to seasonal variations in the hydrology of the area and leachate dilution [54]. During the dry season, for example, reduced rainfall and surface runoff tend to limit the dilution and dispersion of leachates, leading to the accumulation and concentration of wastes in landfill drainage, adjacent water resources, and sediments. The study area is also near to the equator and receives high solar radiation during the dry season. Thus, evaporation should expectedly concentrate the solutes, increasing the values of the measured physicochemical parameters. On the flip side, high levels of precipitation and surface flow during the wet season promote leachate dilution, percolation, and downstream transport of wastes, ions, and PTXEs, resulting in comparatively lower values of the physicochemical parameters. For example, in the Jatibarang landfill (Indonesia), leachate parameters were significantly influenced by seasonal precipitation, except for pH and alkalinity [54].

### 3.2. Spatiotemporal Distribution of PTXEs in Environmental Matrices of KTLF

#### 3.2.1. Water and Leachates

In water, Cr was not detected in samples taken during the wet season, while the concentration of all the other PTXEs ranged from 0.001 ± 0.020 mg/L for Zn sampled from downstream during the wet season to 0.663 ± 0.328 mg/L for Zn sampled from upstream during the dry season (Table 2). There were significant differences among the mean content of PTXEs in the water samples (*H* = 17.0, *p* = 0.03). In the leachates, concentrations of the PTXEs also varied significantly between seasons and the sampled locations (i.e., the clarifier and the two channels) (*H* = 15.18, *p* = 0.009). Only Cr was below the limit of detection (BDL) in the wet season, while the rest of the PTXEs were quantified at concentrations ranging from 0.001 ± 0.010 mg/L for As in the dry season samples from the clarifier and Channel 1 to 0.919 ± 0.661 mg/L for Zn in dry season samples from Channel 1 (Table 3).

The quantified PTXEs (Cu, Cr, and Zn) were at lower concentrations than their WHO permissible limits of 2.00, 0.05, and 3.00 mg/L in potable water [55]. However, As and Pb concentrations were equal to or surpassed their WHO permissible limit of 0.01 mg/L [55]. A recent study of water from borehole and springs within a 5 km radius of KTLF [13] quantified manganese (0.36 mg/L) and Cr (0.278 mg/L) at the highest median concentrations in water, followed by Cu (0.042 mg/L), and Zn (0.008 mg/L), whereas Cd, Pb, cobalt, and As were below detection limits. In contrast, the present study detected comparatively lower concentrations of PTXEs in water, which could result from temporal and spatial variations in the sampling conditions and landfill dynamics. This difference could also be because the study by Aliyinza [13] quantified PTXEs in water using atomic absorption spectroscopy, which generally has higher detection limits than the ICP-OES used in the present study. In a study conducted around waste dumpsites in the Ebonyi, Enugu, and Anambra states of Nigeria, surface water was reported to be contaminated with Pb (0.01–3.16 mg/L), Cu (BDL–2.22 mg/L), and Zn (BDL–0.90 mg/L) [56]. The concentrations of Cr observed in the present study were lower than those reported for landfills in Bangladesh (0.18–2.00 mg/L) [57], Malaysia (0.0032–0.3305 mg/L) [10], and Thailand (0.0147–1.22 mg/L) [58]. Compared to a study conducted in Vientiane, Laos [59], where the concentrations of Cr and Pb in groundwater exceeded WHO permissible limits in both seasons, the corresponding concentrations detected around KTLF were lower and within the guideline values, except at selected sites directly receiving leachate discharge.

For leachates, all the PTXE concentrations reported in the KTLF leachates did not exceed the permissible limits specified by the US EPA for water reuse [60]. An earlier study in KTLF [13] reported median concentrations of 0.02 and 0.17 mg/L for Cu and Zn, which are lower than what is reported in the present study. Another study quantified Cu, Pb, Cr, and Cd with mean concentrations of 51.5, 1.0, 2.4, and 0.8 mg/L, respectively [14]. Sanga et al. [9] quantified Cu (BDL–0.80 mg/L), Pb (0.38–0.59 mg/L), and Zn (5.60–26.10 mg/L) in leachates from municipal solid waste in Iringa, Tanzania. In Tehran, landfill leachates were found to contain Cu (0.08–1.02 mg/L), Cd (0.10–0.32 mg/L), and Pb (0.65–2.17 mg/L) [61].

As discussed for the physicochemical parameters of water and leachate samples, the significantly higher concentrations of PTXEs observed in the dry season compared to the wet season could be attributed to temporal variations in hydrology, leachate chemistry, and dilution. The measured leachate pH values of 8.10 ± 0.16–9.50 ± 0.09 indicate that KTLF leachates are in a mature (methanogenic) stage of decomposition [57]. Mature leachates have neutral to alkaline pH levels due to the degradation of organic acids and the development of buffering from ammonia and carbonate species. They are characteristically anoxic but alkaline, which can alter metal speciation through precipitation, complexation, and reduced mobilization of other elements while maintaining others in solution via complexation with organic ligands [57]. Furthermore, waste scavengers at KTLF wash their salvaged reusable and recyclable materials in the streams [21] which could increase the pollution load of PTXEs in the dry season.

The higher Zn levels observed during the dry season could result from reduced leachate dilution, enhanced evaporation, and concentration effects under limited rainfall, as discussed above. In contrast, the elevated As and Cu concentrations during the wet season could result from increased leaching and the mobilization of soluble metal species from decomposing waste and surface deposits as rainfall and runoff intensify. Under waterlogged conditions, reducing environments tend to favor the release of As from iron oxyhydroxides and the complexation of Cu with dissolved organic matter, thereby increasing their solubility [62].

#### 3.2.2. Superficial Sediments

The quantified PTXEs’ concentrations in sediments ranged from 0.001 ± 0.019 mg/L for As to 6740.858 ± 612.219 mg/L for Cu sampled during the wet and dry seasons, respectively (Table 4). The concentrations of PTXEs in the sediments were highest in the downstream samples, with the dry season samples having Cu and Zn concentrations way above their average shale, toxicity reference, and numerical consensus-based sediment quality guidelines (SQGs) [63,64,65]. Lead concentrations in the dry season sediments from Channel 2 and downstream also exceeded the SQGs, except for the consensus-based probable effect concentration (PEC). In sediments, the SQGs (threshold effect concentration (TEC) and PEC) are used for contamination assessment. In essence, the TEC and PEC are indicated by the effect data’s lower 10th and 50th percentiles [63,65]. Concentration of PTXEs in sediments below their TEC should not result in harmful effects to organisms, while those above PEC are expected to pose health risks [65].

In the sediments sampled from KTLF, the more elevated PTXE concentrations in the dry season samples than those in the wet season could be due to reduced resuspension and oxidation, which favor the retention of metal-bound particulates under low flow conditions [66]. The pH of leachates was slightly acidic to moderately alkaline, which could make them anoxic and facilitate the enrichment of PTXEs in organic rich sediments. Furthermore, changes in sediment geochemistry can lead to the transformation of PTXEs into more bioavailable and sometimes toxic forms [19].

The concentrations of PTXEs in sediment from KTLF and its nearby streams were higher than previously reported in most parts of the world. For example, Adu et al. [58] quantified As, Cu, Cr, and Pb at concentrations of 0.07–0.64 mg/kg in sediments of the Illovu landfill and the Lovu River, South Africa. Ruchuwararak et al. [67] quantified As, Cr, and Pb at mean concentrations of 1.19, 6.97, and 3.20 mg/kg in sediments from a paddy field near a municipal landfill in Thailand. In contrast, a study of landfill and leachate-impacted sediments from the Cisadane River (Indonesia) quantified Cr and Pb at concentrations of 4.29–57.92 and 9.16–72.04 mg/kg, which are comparable to the results obtained for some of the KTLF sediments [68]. Overall, it should be mentioned that the relatively higher concentration of Cr in the sediments observed in the present study could be released back into the water column when physicochemical properties such as redox potential, particle size, temperature, salinity, and pH change [19].

#### 3.2.3. *Colocasia esculenta* Corms

**Figure 3:**
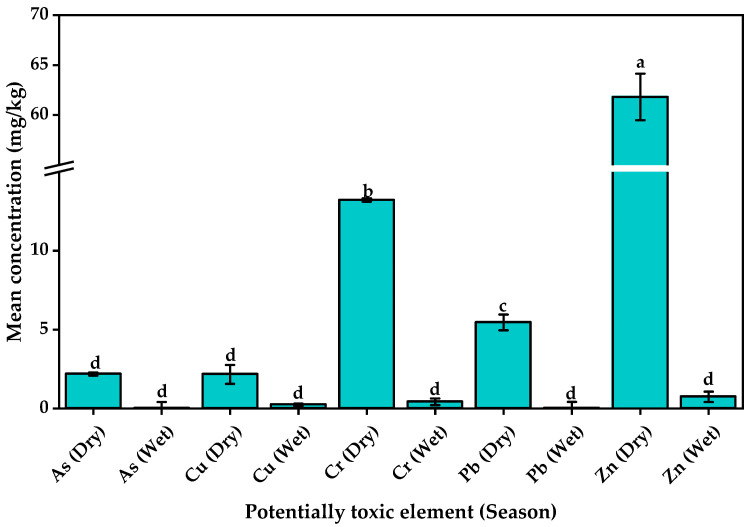
Concentration of potentially toxic elements in *C. esculenta* corms from around Kiteezi landfill. The concentrations of As and Pb during the wet season were 0.010 ± 0.400 mg/kg and 0.018 ± 0.040 mg/kg. Bars with different letters are statistically different (*p* < 0.05).

In this study, *C. esculenta* corms from KTLF contained PTXEs at concentrations ranging from 0.010 ± 0.400 mg/kg for As in wet season corms to 61.822 ± 2.330 mg/kg for zinc in dry season corms (Figure 3). The concentrations of the PTXEs varied significantly between seasons (*p* < 0.05), with the concentration of As (2.187 ± 0.100 mg/kg), Cr (13.223 ± 0.111 mg/kg), Pb (5.462 ± 0.496 mg/kg), and Zn (61.822 ± 2.330 mg/kg) in the dry season corms exceeding their permissible limits of 1.4, 2.3, 5.0, and 60.0 mg/kg [69,70]. These temporal differences could have been introduced by variations in environmental conditions (for example, reduced rainfall and soil moisture during the dry season), which may lead to higher accumulation of PTXEs in plant tissues due to increased soil concentration and decreased dilution. Furthermore, seasonal changes in soil chemistry (such as pH and organic matter content) could influence the individual element’s bioavailability and uptake by the corms [71]. These results indicate that consumption of *C. esculenta* corms harvested in the dry season may pose a higher health risk, and such temporal dynamics should be considered when evaluating food safety in the study area.

*Colocasia esculenta* is known to be a hyperaccumulator [72], but the obtained concentrations of PTXEs in its corms from KTLF were lower than previously quantified in other countries (Table 5). For example, Kundu et al. [72] quantified Zn at concentrations up to 146.67 mg/kg in *C. esculenta* corms from selected industrial sites in Bangladesh. The differences in the concentrations of PTXEs in the corms from KTLF and those previously reported elsewhere may arise from variations in environmental contamination levels, soil properties, agricultural and waste management practices, and industrial activities between the studied regions. For instance, lower levels of industrial discharges, reduced use of agrochemicals, and differences in soil pH and organic matter content at KTLF may have limited metal bioavailability and uptake by the corms. Seasonal factors, sampling times, and botanical varieties (that is, *C. esculenta* var. *esculenta* or *C. esculenta* var. *antiquorum*) were indicated to influence the accumulation of PTXEs in *C. esculenta* corms [73].

### 3.3. Health Risk Assessments from Ingestion of Water and Consumption of C. esculenta

Water had EDI ranging from 1.68 × 10^−8^ mg/kg/day for arsenic to 2.78 × 10^−3^ mg/kg/day for Zn in dry season downstream samples ingested by adults and children, respectively (Table S3). The corresponding hazard quotients were from 5.59 × 10^−5^ to 4.64 × 10^−1^. Interestingly, the hazard indices (1.088 × 10^−3^–5.533 × 10^−1^) were less than 1 in all cases, indicating no potential non-carcinogenic health risks to consumers of water from the studied water sources.

In *C. esculenta*, the EDI ranged from 8.66 × 10^−6^ mg/kg/day for Pb in dry season corms consumed by adults to 9.15 × 10^−1^ for Zn in dry season corms consumed by children (Table S4). The calculated HQ ranged from 6.18 × 10^−3^ for Pb in corms sampled during the wet season consumed by adults to 1.52 × 10^2^ for Zn in dry season corms consumed by children. Overall, the hazard indices calculated (8.48 × 10^−1^–1.89 × 10^2^) showed that only adults who consumed *C. esculenta* corms obtained in the dry season were not likely to experience non-carcinogenic health risks. These health risks would be mostly due to the intake of As, Cr, Pb, and Zn in the contaminated corms.

The cancer risk values due to intake of water were from 7.50 × 10^−9^ for Pb in wet season samples of water from the borehole to 2.52 × 10^−5^ for As in wet season upstream samples in children (Table S5). In adults, the corresponding cancer risk values ranged from 7.13 × 10^−11^ to 1.01 × 10^−6^. The contribution of the PTXEs to cancer risks through this pathway followed the sequence As > Pb > Cr, and the total cancer risks remained within the US EPA acceptable range of 1 × 10^−6^ to 1 × 10^−4^ [79] or were negligible (<1 × 10^−6^). These results indicated that there are non-discernable carcinogenic health risks that may arise from the ingestion of water sourced from the boreholes and Kitetikka stream.

In contrast, the cancer risk values associated with *C. esculenta* corms ranged from 7.36 × 10^−9^ for Pb in wet season corms consumed by adults to 4.85 × 10^−3^ for arsenic in wet season samples consumed by children (Table S5). The carcinogenic health risks were majorly driven by arsenic, followed by Cr and then Pb. The cancer risks due to arsenic (4.85 × 10^−3^ and 1.58 × 10^−3^) and the total cancer risks (1.583 × 10^−3^ and 4.871 × 10^−3^) due to consumption of dry season *C. esculenta* corms were all outside the US EPA range of 1 × 10^−6^ to 1 × 10^−4^. Thus, consumption of *C. esculenta* corms could lead to carcinogenic health effects in its regular consumers.

### 3.4. Heavy Metal Enrichment in Sediments and Associated Ecological Health Risks

The calculated contamination factors ranged from 0.001 for arsenic in sediments collected from the stream as well as Channel 1 during the wet season to 539.3 for Cu sampled from downstream during the dry season (Table 6). Overall, sediments sampled during the dry season tended to have higher contamination factors than those collected during the wet season and followed the order Cu > Zn > Pb > Cr > As.

**Figure 4:**
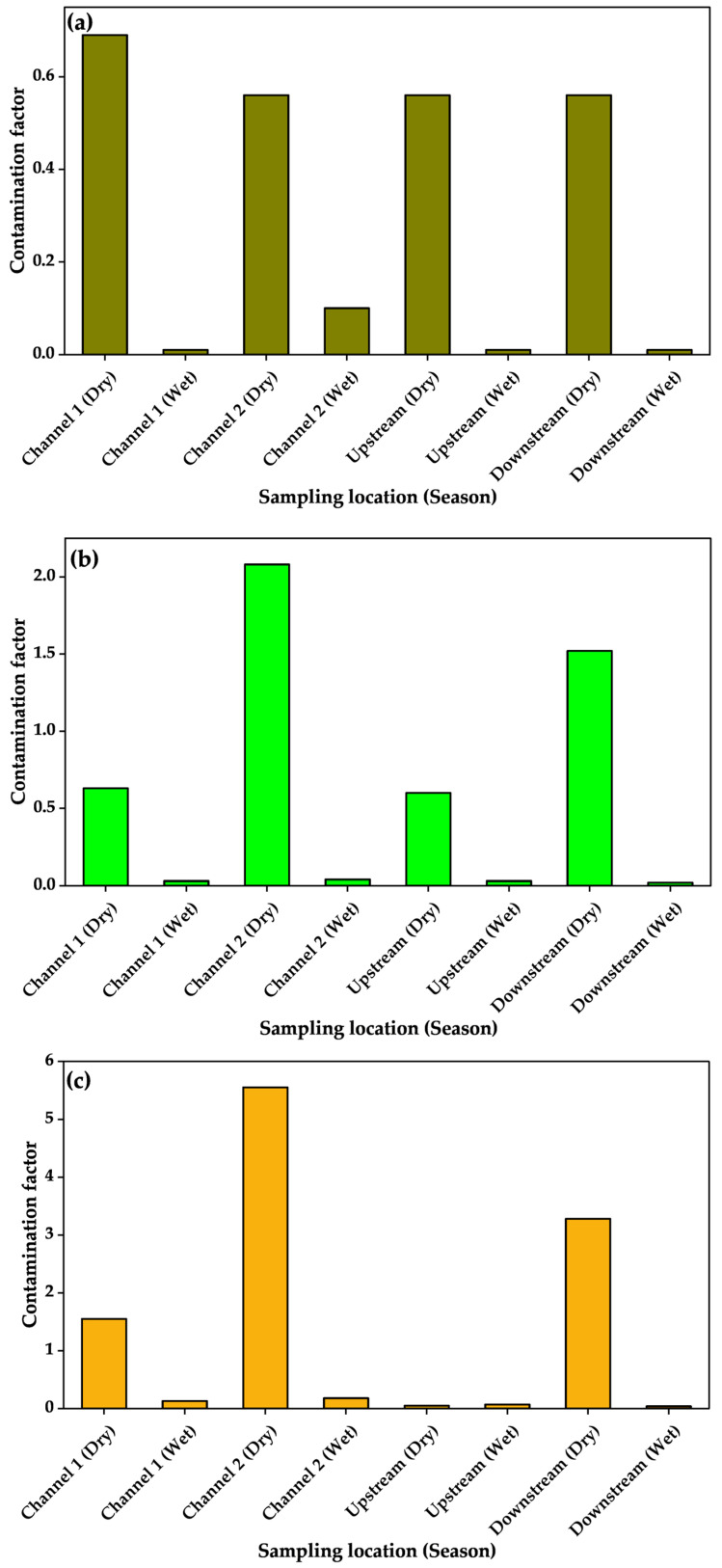
Contamination factors of carcinogenic elements in sediments from Kiteezi landfill: (**a**) arsenic, (**b**) chromium, and (**c**) lead.

**Figure 5:**
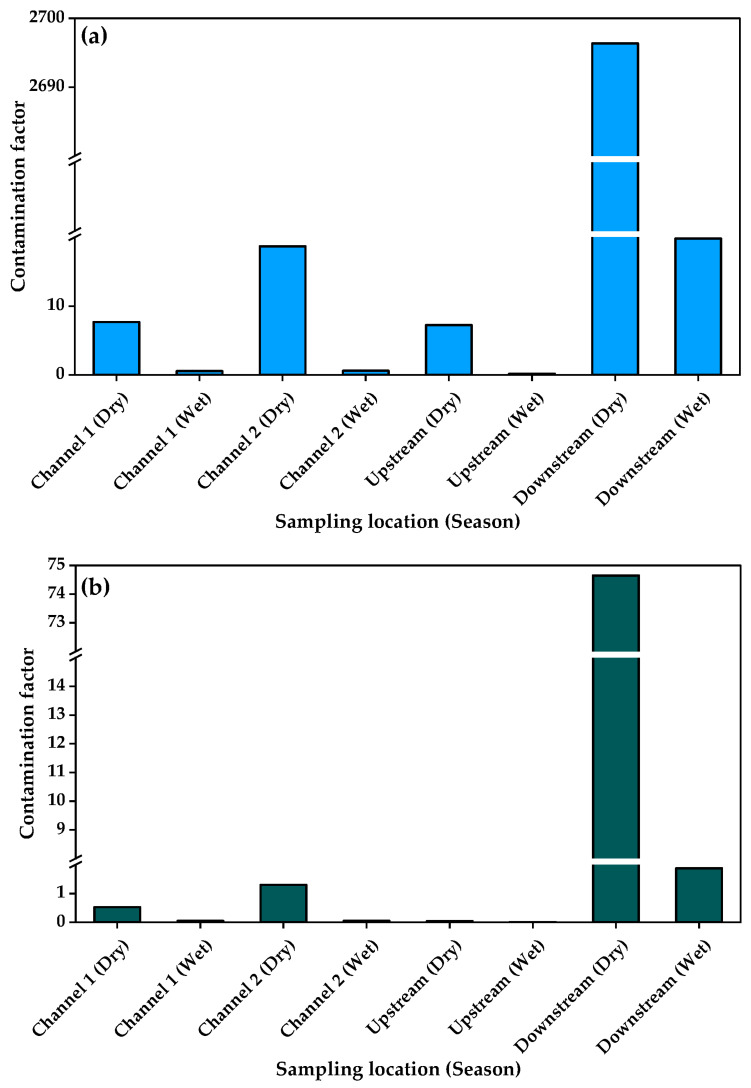
Contamination factors of non-carcinogenic elements in sediments from Kiteezi landfill: (**a**) copper and (**b**) zinc.

**Figure 6:**
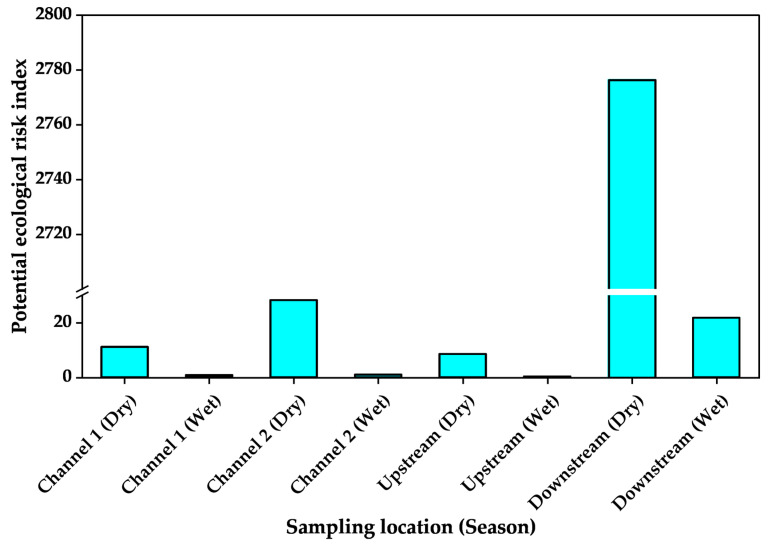
Mean potential ecological risk index of potentially toxic elements in sediments from Kiteezi landfill and its vicinity.

The PLI calculated from the product of the individual metal CF per sampling site ranged from 0.01 for upstream samples taken during the wet season to 4.07 for downstream samples taken during the dry season. Thus, only the dry season downstream sediments had PLI > 1. When the index of geoaccumulation was calculated, the values ranged from −11.4 for arsenic in wet season samples to 8.5 for Cu in sediments sampled from downstream during the dry season. The ecological risk coefficients of the PTXEs ranged from 0.01 for arsenic in wet season samples (Figure 4) and Zn in wet season upstream sediments to 2696.34 for Cu in sediments sampled from downstream during the dry season (Figure 5). The computed potential ecological risk indices ranged from 0.30 for upstream sediments sampled during the wet season to 2776.36 for downstream sediments sampled during the dry season (Figure 6).

Enrichment of PTXEs in the sedimentary phase of streams and associated water sources in the vicinity of landfills is expected due to the diverse nature of municipal waste. From the indices calculated in the present study, the contamination factors showed low contamination (CF < 1), moderate contamination (1 ≤ CF < 3), and very high contamination (CF > 6 for Cu and Zn) when Hakanson’s criteria [38] were used to interpret the values obtained. These results indicated strong enrichment of Cu and Zn downstream, which was further supported by the PLI for the same point being fourfold greater than 1 [80]. The observed order of contamination factors (Cu > Zn > Pb > Cr > As) in the sediments stems from differences in the sources of the PTXEs, their geochemical behavior, and affinity for sediment components. Copper and Zn are among the most abundant metal elements in municipal solid waste due to their widespread use in electrical components, alloys, and packaging materials [81], which may explain their relatively higher enrichment. Both elements should thus exhibit moderate mobility under the alkaline and reducing conditions typical in KTLF leachates (Table 1), promoting their transfer to sediments. Lead is less mobile but can accumulate through adsorption onto organic matter and iron–manganese oxides [82]. For Cr and As, the lowest contamination factors could be due to their stronger association with mineral phases and lower solubility under the prevailing near-neutral to alkaline pH of KTLF leachates, which favors their precipitation [83].

The I_geo_ calculated for downstream samples was greater than 5, which corresponds to extreme Cu and Zn contamination (class 6) [37] during the dry season. Assessment of ecological risks indicated mostly low contamination. However, downstream samples exhibited high contamination. These results indicate that Cu and Zn are being enriched in sediments from streams near KTLF and could pose ecological risks to biodiversity in and around KTLF.

## 4. Conclusions

Potentially toxic elements were found to have been enriched in matrices sampled from KTLF and its surroundings during the dry season. The heavy metal concentrations decreased during the wet season, which could be attributed to dilution effects or leaching into the surrounding environment. Human health and ecological and pollution risk indices suggested a potential threat to both humans and the ecosystem. These findings highlight the urgent need for improved landfill management strategies in Uganda to mitigate pollution, and they also emphasize the need that any replacement for KTLF should be properly managed to prevent potential environmental pollution.

## Figures and Tables

**Table 1:** Temporal variation in physicochemical parameters in environmental matrices of Kiteezi landfill, Uganda.

Matrices	Season	pH	Electrical Conductivity (μS/cm)	Temperature (°C)	Oxidation–Reduction Potential (mV)
Water (Borehole)	Dry	5.33 ± 0.10 ^a^	233.8 ± 1.2 ^a^	24.6 ± 2.4 ^a^	78.5 ± 0.5 ^a^
	Wet	5.30 ± 0.04 ^a^	213.0 ± 17.0 ^a^	23.8 ± 4.7 ^a^	78.5 ± 4.9 ^a^
Water (Upstream)	Dry	6.76 ± 0.02 ^b^	225.0 ± 14.1 ^a^	24.3 ± 1.3 ^a^	−4.0 ± 2.8 ^b^
	Wet	6.91 ± 0.01 ^b^	195.0 ± 7.1 ^a^	20.6 ± 0.4 ^a^	−13.5 ± 0.7 ^b^
Water (Downstream)	Dry	8.20 ± 0.10 ^c^	11,650.0 ± 338.1 ^b^	24.3 ± 1.0 ^a^	−92.0 ± 1.4 ^c^
	Wet	8.50 ± 0.10 ^c,d^	8550.0 ± 3111.3 ^b,c^	23.9 ± 4.5 ^a^	−110.0 ± 4.2 ^d^
Leachate (Clarifier)	Dry	9.30 ± 0.05 ^e^	10,046.7 ± 321.5 ^b,c^	23.9 ± 0.4 ^a^	−158.5 ± 0.7 ^e^
	Wet	9.50 ± 0.09 ^e^	7240.0 ± 183.8 ^b,c^	24.1 ± 4.6 ^a^	−168.0 ± 5.7 ^e^
Leachate (Channel 1)	Dry	8.10 ± 0.16 ^d^	28,966.7 ± 907.4 ^d^	24.2 ± 0.8 ^a^	−87.0 ± 1.4 ^c^
	Wet	8.60 ± 0.11 ^c,d^	26,400.0 ± 424.3 ^d^	23.7 ± 4.2 ^a^	−110.0 ± 4.2 ^d^
Leachate (Channel 2)	Dry	8.49 ± 0.03 ^c,d^	27,133.3 ± 1050.4 ^d^	24.0 ± 1.2 ^a^	−106.5 ± 0.7 ^d^
	Wet	8.65 ± 0.07 ^c,d^	22,450.0 ± 353.6 ^e^	23.8 ± 4.4 ^a^	−113.0 ± 4.2 ^d^

Indicated values are means ± standard deviations of triplicates. Means with different letters in a column are statistically different as per Tukey test (*p* < 0.05) or Dunn’s test (for oxidation–reduction potential).

**Table 2:** Concentration of potentially toxic elements (mg/L) in water from Kiteezi landfill and its vicinity.

Source	Season	As	Cu	Cr	Pb	Zn
Borehole	Dry	0.010 ± 0.009 ^a^	0.007 ± 0.004 ^a^	0.008 ± 0.002 ^a^	0.021 ± 0.003 ^a^	0.210 ± 0.043 ^a^
	Wet	0.037 ± 0.027 ^b^	0.018 ± 0.002 ^a^	<0.001	0.005 ± 0.003 ^b^	0.002 ± 0.001 ^b^
Upstream	Dry	0.009 ± 0.001 ^a^	0.003 ± 0.001 ^b^	0.007 ± 0.003 ^a^	0.028 ± 0.010 ^a^	0.023 ± 0.015 ^c^
	Wet	0.040 ± 0.038 ^b^	0.115 ± 0.067 ^c^	<0.001	0.051 ± 0.031 ^c^	0.003 ± 0.002 ^b^
Downstream	Dry	0.001 ± 0.050 ^c^	0.048 ± 0.019 ^d^	0.072 ± 0.012 ^b^	0.175 ± 0.115 ^d^	0.663 ± 0.328 ^d^
	Wet	0.022 ± 0.008 ^a^	0.025 ± 0.014 ^e^	<0.001	0.015 ± 0.011 ^a^	0.001 ± 0.020 ^b^
WHO limits [55]		0.01	2.0	0.05	0.01	3.0

Note: Indicated values are means ± standard deviations of replicates. Means with different letters in a column are statistically different (*p* < 0.05).

**Table 3:** Concentration of potentially toxic elements (mg/L) in leachates from Kiteezi landfill.

Source	Season	As	Cu	Cr	Pb	Zn
Clarifier	Dry	0.011 ± 0.010 ^a^	0.016 ± 0.002 ^a^	0.040 ± 0.001 ^a^	0.009 ± 0.001 ^a^	0.184 ± 0.032 ^a^
	Wet	0.123 ± 0.103 ^b^	0.035 ± 0.022 ^a^	<0.001	0.032 ± 0.030 ^b^	0.004 ± 0.003 ^b^
Channel 1	Dry	0.001 ± 0.010 ^c^	0.101 ± 0.015 ^b^	0.172 ± 0.012 ^b^	0.092 ± 0.017 ^c^	0.778 ± 0.263 ^c^
	Wet	0.057 ± 0.032 ^d^	0.082 ± 0.056 ^b^	<0.001	0.090 ± 0.070 ^c^	0.016 ± 0.014 ^d^
Channel 2	Dry	0.003 ± 0.002 ^c^	0.091 ± 0.001 ^b^	0.112 ± 0.025 ^c^	0.099 ± 0.031 ^c^	0.919 ± 0.661 ^e^
	Wet	0.126 ± 0.046 ^b^	0.097 ± 0.040 ^b^	<0.001	0.082 ± 0.021 ^c^	0.003 ± 0.002 ^b^

Note: Indicated values are means ± standard deviations of triplicates. Means with different letters in a column are statistically different (*p* < 0.05).

**Table 4:** Concentration of potentially toxic elements (mg/kg) in sediments from Kiteezi landfill compared against sediment quality guidelines.

Source	Season	As	Cu	Cr	Pb	Zn
Channel 1	Dry	0.125 ± 0.025 ^a^	19.176 ± 18.133 ^a^	18.857 ± 17.645 ^a^	17.104 ± 16.394 ^a^	37.279 ± 36.029 ^a^
	Wet	0.001 ± 0.040 ^b^	1.407 ± 0.233 ^b^	1.013 ± 0.182 ^b^	1.421 ± 0.239 ^b^	3.606 ± 0.473 ^b^
Channel 2	Dry	0.100 ± 0.001 ^a^	46.705 ± 6.410 ^c^	62.543 ± 20.907 ^c^	61.099 ± 23.713 ^c^	90.803 ± 30.060 ^c^
	Wet	0.018 ± 0.011 ^c^	1.489 ± 0.321 ^b^	1.276 ± 0.121 ^b^	1.998 ± 0.499 ^d^	3.294 ± 0.688 ^b^
Upstream	Dry	0.100 ± 0.060 ^a^	18.093 ± 16.206 ^a^	18.024 ± 19.067 ^a^	0.537 ± 0.197 ^a^	2.923 ± 30.324 ^b^
	Wet	0.001 ± 0.020 ^b^	0.423 ± 0.077 ^b^	1.038 ± 0.163 ^b^	0.812 ± 0.207 ^a^	0.879 ± 0.179 ^d^
Downstream	Dry	0.100 ± 0.033 ^a^	6740.858 ± 612.219 ^d^	45.71 ± 4.025 ^c^	36.078 ± 9.411 ^e^	5225.724 ± 322.895 ^e^
	Wet	0.001 ± 0.019 ^b^	49.500 ± 11.892 ^e^	0.522 ± 0.229 ^b^	0.483 ± 0.024 ^a^	131.500 ± 39.955 ^f^
Overall range		0.001–0.125	0.423–6740.858	0.522–62.543	0.483–61.099	0.879–5225.724
Average shale value [63]		13	45	90	20	95
Toxicity reference value [64]		8.2	16	26	31	110
Threshold effect concentration [65]		9.79	31.6	43.4	35.8	121
Probable Effect Concentration [65]		33.0	149	111	128	459

Note: Indicated values are mean ± standard deviation of triplicates. Means with different letters in a column are statistically different (*p* < 0.05).

**Table 5:** Concentration of potentially toxic elements in *C. esculenta* corms around Kiteezi landfill in comparison with global reports.

Location	As	Cu	Cr	Pb	Zn	References
Kiteezi landfill, Uganda	0.001–2.187	0.237–2.167	0.423–13.223	0.018–5.462	0.741–61.822	Current study
Atiwa East, Ghana	0.43–1.89	12.78–25.12	9.11–16.45	2.14–6.33	–	Cobbinah et al. [74]
Industrial areas, Bangladesh	–	4.43–266.67	1.80–19.33	BDL–6.33	3.8–146.67	Kundu et al. [72]
Kenya	BDL–0.19	–	3.02–3.90	1.05–1.08	8.1 –11.3	Uwamariya et al. [75]
Lake Victoria Basin (Tanzania, Uganda, Kenya)	BDL–3.5	3.0–7.5		BDL–6.0	–	Mongi and Chove [76]
Tarkwa, Ghana	366 and 401	–	–	–	–	Essumang et al. [77]
Kade, Ghana	0.012–0.019	–	–	–	–	Adjei-Mensah et al. [78]
Maximum allowed concentration	1.4	40	2.3	5	60	[69,70]

Note: BDL = below method detection limit; – not studied.

**Table 6:** Contamination assessment indices for sediments from Kiteezi landfill and its vicinity.

Sampling Point	Season	As		Cu		Cr		Pb		Zn		PLI
		CF	I_geo_	CF	I_geo_	CF	I_geo_	CF	I_geo_	CF	I_geo_	
Channel 1	Dry	0.07	−4.4	1.5	0.03	0.3	−2.3	0.3	−2.3	0.5	−1.5	0.40
	Wet	0.001	−11.4	0.1	−3.7	0.02	−6.5	0.03	−5.9	0.1	−4.9	0.02
Channel 2	Dry	0.1	−4.8	3.7	1.3	1.0	−0.5	1.1	−0.4	1.3	−0.2	0.80
	Wet	0.01	−7.2	0.1	−3.7	0.02	−6.1	0.04	−5.4	0.1	−5.0	0.03
Upstream	Dry	0.1	−4.8	1.5	−0.1	0.3	−2.3	0.01	−7.3	0.04	−5.2	0.10
	Wet	0.001	−11.4	0.03	−5.5	0.02	−6.4	0.01	−6.7	0.01	−6.9	0.01
Downstream	Dry	0.1	−4.8	**539.3**	8.5	0.8	−1.0	0.7	−1.2	**74.7**	5.6	4.07
	Wet	0.001	−11.4	4.0	1.4	0.01	−7.4	0.01	−7.4	1.9	0.3	0.05

Note: Contamination factors (CF) in **bold** indicate very high levels of sediment contamination according to Hakanson’s criteria [38].

## Data Availability

The original contributions presented in this study are included in the article/Supplementary Material. Further inquiries can be directed to the corresponding authors.

## Supplementary Materials

The following supporting information can be downloaded at: https://www.mdpi.com/article/10.3390/jox15060185/s1, Table S1. Exposure factors used in health risk assessments due to potentially toxic elements in water and *C. esculenta* from Kiteezi landfill, Uganda; Table S2. Classification values of pollution and risk indices used in the study of potentially toxic elements in sediments from Kiteezi landfill, Uganda; Table S3. Estimated daily intakes, target hazard quotients, and hazard indices from ingestion of potentially toxic elements in water from around Kiteezi landfill, Uganda; Table S4. Estimated daily intakes, target hazard quotients, and hazard indices from consumption of contaminated *C. esculenta* corms; Table S5. Cancer risk and total cancer risk values due to ingestion of water and consumption of *C. esculenta* corms. Refs. [27,37,38,84] are cited in Supplementary Materials file.
