# Supplementary material for: Ecological and Human Health Risks from Potentially Toxic Elements in Environmental Matrices of Kiteezi Landfill, Uganda
Source: J Xenobiot. 2025 Nov 4;15(6):185. doi: 10.3390/jox15060185 (PMC12641813; doi:10.3390/jox15060185)
Supplement: Supplementary file 1 [file jox-15-00185-s001.zip › jox-3934714-supplementary.pdf]

Supplementary Materials: Ecological and Human Health Risks from Potentially Toxic Elements in Environmental Matrices of Kiteezi

Table S1. Exposure factors used in health risk assessments due to potentially toxic elements in water and *C. esculenta* from Kiteezi landfill, Uganda

Factor	Description (units)	Children	Adults
C_{hm}	Measured elements' concentration (mg/L or mg/kg)	This study	This study
F*i*	Ingestion rate (L/day or g/person/day)	1.8 (water), 231.5 (*C. esculenta*)	2.1 (water), 301.0 (*C. esculenta*)[1]
E*x*F*r*	Exposure frequency (days/year)	350	350
E*x*D*r*	Exposure duration (years)	6	30
W_{ab}	Body weight (kg)	15	60 [2]
T*a*	Average time (days)	Non-carcinogenic metals: E_xD_r × 365 days/year; carcinogenic metals: 60 years × 365 days/year	

Table S2. Classification values of pollution and risk indices used in study of potentially toxic elements in sediments from Kiteezi landfill, Uganda

Pollution and risk indicators	Classification values	Description
Contamination factor (CF) [3]	CF< 1	Low contamination
	1 ≤CF< 3	Moderate contamination
	3 ≤ CF< 6	Considerable contamination
	CF> 6	Very high contamination
Geo-accumulation index (I_{geo}) [4]	I_{geo}<0 (class 0)	Practically uncontaminated
	0<I_{geo}<1 (class 1)	Low to median contamination
	1<I_{geo}<2 (class 2)	Median contamination
	2<I_{geo} <3 (class 3)	Median to strong contamination
	3<I_{geo}<4 (class 4)	Serious contamination
	4<I_{geo}< 5 (class 5)	Serious to extreme contamination
	I_{geo}> 5 (class 6)	Extreme contamination
Pollution Load index (PLI) [2]	PLI < 1	Unpolluted
	PLI > 1	Polluted
Ecological risk (E_i and RI) [3]	E_i < 40; RI < 95	Low contamination
	40 ≤ E_i ≤ 80; 95 ≤ RI ≤ 190	Moderate contamination
	80 ≤ E_i ≤ 160; 190 ≤ RI ≤ 380	Considerable contamination
	160 ≤ E_i ≤ 320; RI ≥ 380	High contamination
	320 ≤ E_i	Very high contamination

Table S3. Estimated daily intakes, target hazard quotients and hazard indices from ingestion of potentially toxic elements in water from around Kiteezi landfill, Uganda

Index	Age group	Season	As	Cu	Cr	Pb	Zn	Hazard index
Estimated daily intake (mg/kg/day)	Children	Dry	4.20E-06	2.94E-05	3.36E-06	8.82E-06	8.82E-04	–
		Wet	1.55E-05	7.56E-05	–	2.10E-06	8.40E-06	–
		Dry	3.78E-06	1.26E-05	2.94E-06	1.18E-05	9.66E-05	–
		Wet	1.68E-05	4.83E-04	–	2.14E-05	1.26E-05	–
		Dry	4.20E-07	2.02E-04	3.02E-05	7.35E-05	2.78E-03	–
		Wet	9.24E-06	1.05E-04	–	6.30E-06	4.20E-06	–
Target hazard quotient		Dry	1.40E-02	7.35E-04	3.36E-03	6.30E-03	1.47E-01	1.714E-01
		Wet	5.18E-02	1.89E-03	–	1.50E-03	1.40E-03	5.659E-02
		Dry	1.26E-02	3.15E-04	2.94E-03	8.40E-03	1.61E-02	4.036E-02
		Wet	5.60E-02	1.21E-02	–	1.53E-02	2.10E-03	8.548E-02
		Dry	1.40E-03	5.04E-03	3.02E-02	5.25E-02	4.64E-01	5.533E-01
		Wet	3.08E-02	2.63E-03	–	4.50E-03	7.00E-04	3.863E-02
Estimated daily intake (mg/kg/day)	Adults	Dry	1.68E-07	2.35E-07	1.34E-07	3.52E-07	7.05E-06	–
		Wet	6.21E-07	6.04E-07	–	8.39E-08	6.71E-08	–
		Dry	1.51E-07	1.01E-07	1.17E-07	4.70E-07	7.72E-07	–
		Wet	6.71E-07	3.86E-06	–	8.56E-07	1.01E-07	–
		Dry	1.68E-08	1.61E-06	1.21E-06	2.94E-06	2.23E-05	–
		Wet	3.69E-07	8.39E-07	–	2.52E-07	3.36E-08	–
Target hazard quotient		Dry	5.59E-04	5.87E-06	1.34E-04	2.52E-04	1.17E-03	2.126E-03
		Wet	2.07E-03	1.51E-05	–	5.99E-05	1.12E-05	2.156E-03
		Dry	5.03E-04	2.52E-06	1.17E-04	3.36E-04	1.29E-04	1.088E-03
		Wet	2.24E-03	9.65E-05	–	6.11E-04	1.68E-05	2.962E-03
		Dry	5.59E-05	4.03E-05	1.21E-03	2.10E-03	3.71E-03	7.111E-03
		Wet	1.23E-03	2.10E-05	–	1.80E-04	5.59E-06	1.437E-03

Note: – means Not applicable.

Table S4. Estimated daily intakes, target hazard quotients and hazard indices from consumption of contaminated *C. esculenta* corms

Index	Age group	Season	As	Cu	Cr	Pb	Zn	Hazard index
Estimated daily intake (mg/kg/day)	Children	Dry	3.24E-03	3.21E-02	1.96E-02	8.08E-03	9.15E-01	–
		Wet	1.48E-05	3.51E-03	6.26E-04	2.66E-05	1.10E-02	–
	Adults	Dry	1.05E-03	1.04E-02	6.36E-03	2.63E-03	2.97E-01	–
		Wet	4.81E-06	1.14E-03	2.03E-04	8.66E-06	3.56E-03	–
Target hazard quotient	Children	Dry	1.08E+01	8.02E-01	1.96E+01	5.77E+00	1.52E+02	1.89E+02
		Wet	4.93E-02	8.77E-02	6.26E-01	1.90E-02	1.83E+00	2.61E+00
	Adults	Dry	3.51E+00	2.61E-01	6.36E+00	1.88E+00	4.96E+01	6.16E+01
		Wet	1.60E-02	2.85E-02	2.03E-01	6.18E-03	5.94E-01	8.48E-01

Note: Hazard indices in red are above 1, indicating likeliness of non-carcinogenic effects.

Table S5. Cancer risk and total cancer risk values due to ingestion of water and consumption of *C. esculenta* corms

Exposure source	Season	Children				Adults			
		As	Cr	Pb	Total cancer risk	As	Cr	Pb	Total cancer risk
Water (Borehole)	Dry	6.30E-06	1.68E-09	7.50E-09	6.31E-06	2.52E-07	6.712E-11	3.00E-10	2.52E-07
	Wet	2.33E-05	–	1.79E-09	2.33E-05	9.31E-07	–	7.13E-11	9.31E-07
Water (Upstream)	Dry	5.67E-06	1.47E-09	1.00E-08	5.68E-06	2.27E-07	5.873E-11	3.99E-10	2.27E-07
	Wet	2.52E-05	–	1.82E-08	2.52E-05	1.01E-06	–	7.27E-10	1.01E-06
Water (Downstream)	Dry	6.30E-07	1.512E-08	6.25E-08	7.08E-07	2.52E-08	6.041E-10	2.50E-09	2.83E-08
	Wet	1.39E-05	–	5.36E-09	1.39E-05	5.54E-07	–	2.14E-10	5.54E-07
C. esculenta	Dry	4.85E-03	9.78E-06	6.87E-06	4.871E-03	1.58E-03	3.18E-06	2.23E-06	1.583E-03
	Wet	2.22E-05	3.13E-07	2.26E-08	2.253E-05	7.22E-06	1.02E-07	7.36E-09	7.325E-06

Note: Cancer risks in red are higher than the 1×10^{-4}, indicating likeliness of carcinogenic health risks; – means Not applicable.

References

1. Omara, T.; Karungi, S.; Kalukusu, R.; Nakabuye, B.; Kagoya, S.; Musau, B. Mercuric pollution of surface water, superficial sediments, Nile tilapia (*Oreochromis nilotica* Linnaeus 1758 [Cichlidae]) and yams (*Dioscorea alata*) in auriferous areas of Namukombe stream, Syanyonja, Busia, Uganda. *PeerJ* **2019**, *7*, e7919.
2. Rutehenda, D.R.; Adaku, C.; Omara, T.; Angiro, C.; Ntambi, E. Enrichment, Bioaccumulation and Health Risks of Trace Metals in Soils and Leafy Vegetables Grown on the Banks of the Ugandan Lifeline River, River Rwizi. *World* **2024**, *5*, 136-154.
3. Hakanson, L. An ecological risk index for aquatic pollution control. A sedimentological approach. *Water Res.* **1980,** *14,* 975–1001.
4. Müller, G. Die Schwermetallbelastung der Sedimenten des Neckars und Seiner Nebenflüsse. *Chemiker-Zeitung*. **1981**, *6,* 157–164.
